# Establishment and Validation of a C57BL/6J Mouse Model for Melasma

**DOI:** 10.1111/cpr.70078

**Published:** 2025-07-10

**Authors:** Wenzhu Wang, Xiaojie Sun, Yunyao Liu, Yin Yang, Hedan Yang, Xiaoli Zhang, Xiuzhen Li, Haoxiang Xu, Xu Chen, Tong Lin

**Affiliations:** ^1^ Department of Laser, Hospital for Skin Diseases, Institute of Dermatology Chinese Academy of Medical Sciences & Peking Union Medical College Nanjing China; ^2^ Jiangsu Key Laboratory of Molecular Biology for Skin Diseases and STIs Department, Hospital for Skin Diseases Institute of Dermatology, Chinese Academy of Medical Sciences & Peking Union Medical College Nanjing China

**Keywords:** C57BL/6J mice, melasma‐like mouse model, oxidative stress, pigmentation

## Abstract

Melasma is a recurrent and treatment‐resistant hyperpigmentation disorder characterized by a complex and multifactorial pathogenesis. However, the lack of a stable and reliable animal model has hindered systematic investigations into its onset and progression. In this study, we established a melasma‐like model in C57BL/6J mice by combining broadband UVB irradiation, intramuscular progesterone administration, and induced emotional stress. The affected skin areas exhibited irregular, brown hyperpigmented patches. Histopathological analysis revealed an accumulation of melanin granules in the epidermis and superficial dermis, elevated levels of tyrosinase (TYR) in both skin and plasma, systemic oxidative stress imbalance, and reduced autophagic activity in the lesional skin. Furthermore, this model displayed distinct differences from a UV‐induced post‐inflammatory hyperpigmentation (PIH) model. Notably, the melasma‐like mice responded to tranexamic acid treatment in a manner that closely resembled clinical outcomes observed in human patients. Collectively, these findings establish a stable, reproducible, and clinically relevant mouse model of melasma, providing a valuable platform for future research into its pathogenesis and treatment.

## Graphical abstract

A melasma‐like mouse model was established in C57BL/6J mice using a combination of UVB irradiation, progesterone administration, and chronic psychological stress. This model replicates key clinical features and biomarker alterations observed in human melasma. Importantly, it is fundamentally distinct from UV‐induced post‐inflammatory hyperpigmentation (PIH) and exhibits a therapeutic response to tranexamic acid treatment.

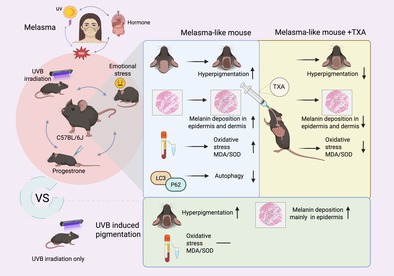

## 1 Introduction

Melasma primarily affects women aged 30–40 with Fitzpatrick skin types III‐IV, manifesting as irregular brown patches on sun‐exposed facial areas. It often recurs, significantly impacting both mental and physical well‐being [1, 2, 3]. Its pathogenesis is driven by complex interactions between ultraviolet (UV) light, hormones and environmental factors. Among enviromental factors, UVR is a primary driver of melasma oneset and progression, inducing abberant melanogenesis, oxidative stress, skin barrier dysfunction, and lipid metabolism dysregulation, all of which contribute to its chronic development [4, 5]. Endocrine hormones also play a key role in melanin regulation [6]. Oestrogen (such as 17β‐estradiol) and progesterone, involved in pregnancy, oral contraceptives and hormone replacement therapy, are thought to regulate melanin synthesis via non‐classical membrane‐bound receptors [7, 8, 9]. These hormones may further stimulate melanogenic enzymes such as TYR and interact with skin hormone receptors, thereby amplifying melanin production [10]. Oxidative stress is another critical factor in melasma. Environmental stressors, including UV exposure, pollution and sleep deprivation, generate reactive oxygen species (ROS) in the skin, with malondialdehyde (MDA) as an end product, whilst antioxidants like superoxide dismutase (SOD) and glutathione help maintain balance [11]. Studies have found that patients with melasma often exhibit abnormal oxidant‐antioxidant levels in their serum, which correlate with disease severity [12, 13, 14, 15].

Melasma is currently recognized as a disorder with a complex pathogenesis involving multiple factors, including disruption of the skin barrier, paracrine signalling abnormalities, fibroblast senescence and basement membrane damage, all of which contribute to increased melanogenesis [16, 17]. Amongst these, melanocyte hyperactivity is considered the central mechanism underlying the persistent overproduction of melanin, which accounts for the chronic and relapsing nature of melasma [18]. Melanin synthesis is regulated by a network of genes and signalling pathways [19, 20]; however, the lack of reliable biomarkers continues to hinder accurate diagnosis and targeted treatment strategies. Recent studies have highlighted the critical role of autophagy in regulating pigmentation associated with melasma. Notably, reduced autophagic activity has been observed in keratinocytes, melanocytes and upper dermal fibroblasts within melasma lesions [21, 22]. Additionally, the expression of autophagy‐related proteins such as LC3 is significantly decreased in affected skin, contributing to premature skin aging, compromised skin barrier function and impaired melanosome degradation, which collectively result in sustained melanin accumulation [23].

Owing to the incomplete understanding of melasma's underlying pathogenesis, existing therapeutic approaches—such as topical formulations and oral tranexamic acid—often yield suboptimal results, lacking sustained and consistent efficacy [24]. Currently, there is no dependable animal model for melasma; although studies have tried inducing melasma through UV exposure and progesterone injections, these methods have yet to achieve consistent experimental validation. Establishing a suitable animal model could be instrumental in elucidating melasma's underlying mechanisms and advancing more effective treatments. Recognising the significant links between melasma and factors like UV radiation, sex hormones and endocrine influences, our research team developed a melasma model in mice using a combination of UV exposure, intramuscular progesterone and stress induction. After careful comparison, C57BL/6J mice were selected as the preferred model organism for this study [25].

This study aims to investigate changes in melasma‐induced mice in terms of skin appearance, histopathology, melanin synthesis and oxidative stress, to compare these findings with those from UV‐induced post‐inflammatory hyperpigmentation and to compare local autophagy levels and other pathogenesis‐related mechanisms associated with melasma. Additionally, it assesses the response to tranexamic acid, providing a suitable mouse model for advancing melasma research and treatment.

## 2 Materials and Methods

### 2.1 Animals

To establish the melasma mouse model, we initially used specific pathogen‐free (SPF) C57BL/6J mice (*n* = 30, female, aged 6–8 weeks, weighing 18–22 g). For verification and comparisons with the post‐inflammatory pigmentation model and the tranexamic acid administration model, an additional group of SPF C57BL/6J mice (*n* = 80, female, aged 6–8 weeks, weighing 18–21 g) was included. These mice were sourced from Jiangsu Jicui Yaokang Biotechnology Company and fed a standard animal diet. All mice were housed according to the animal experimentation guidelines established by the Experimental Animal Center of the Skin Hospital of the Chinese Academy of Medical Sciences. Environmental conditions were maintained at a temperature of 22°C ± 2°C, relative humidity of 50% ± 10% and a 12‐hour light–dark cycle, with unrestricted access to standard laboratory chow and water. The experiment received approval from the Medical Ethics Committee of the Dermatology Hospital of the Chinese Academy of Medical Sciences (Approval Number: 2023‐dw‐014). The relevant supporting documents have been uploaded in Supplement2.

### 2.2 Materials and Reagents

Key equipment included a UV irradiator and a UVB intensity detector, both supplied by Shanghai Sigma High Tech Co. Ltd. (220 V/50 Hz/≤ 1400VA). Paraffin embedding and tissue section staining services were provided by Wuhan Pinuofei Biotechnology Co. Ltd. and Nanjing Frith Biotechnology Co. Ltd. The Masson–Fontana melanin staining solution was sourced from Proteinbio, Nanjing, China. Kits for detecting total SOD activity (WST‐8 method) were purchased from Shanghai Biyuntian Biotechnology, whilst the mouse TYR kit (ELISA) and mouse MDA quantitative detection kit (ELISA) were obtained from Shanghai Enzyme Linked Biological.

### 2.3 Methods

#### 2.3.1 Modelling Melasma in Mice

After a week of adaptation, mice were randomly divided into three groups: a normal control group, Mel1 and Mel2. Each of the Mel1 and Mel2 groups was further split equally into two subgroups: one for head modelling and the other for ear modelling. Prior to the start of modelling, a mild hair removal cream was used to remove hair from the designated areas. Modelling was conducted over 28 consecutive days, with hair removal repeated every 5–7 days as needed. To reduce the impact of hair removal on UV exposure, UV irradiation was administered 12 h after hair removal. For the head modelling group, the shaved area was marked and measured (approximately 1.5 x 1.5 cm), and non‐exposed areas were shielded with tin foil. In the ear modelling group, both ears were depilated, with only the left ear exposed to UVB, while the right ear and rest of the body were protected. The normal control group received only depilation and anaesthesia, with no additional treatments. The melasma model groups (Mel1 and Mel2) underwent combined treatments of UVB irradiation, intramuscular progesterone injection and emotional stress. The head modelling group was exposed to UVB (290–320 nm wavelength) at a dose of 30 mJ/cm^2^ daily, while the ear modelling group received a dose of 60 mJ/cm^2^ on the dorsal side of the left ear. Progesterone was administered intramuscularly at different concentrations in the Mel1 and Mel2 groups. Additionally, mice in the model groups were subjected to daily emotional stressors, including restricted access to food and water, tail suspension by clamping, and physical restraint. These combined modelling procedures were maintained for 28 days, during which pigmentation changes were regularly observed and documented through photographs.

#### 2.3.2 UVB‐Induced Post‐Inflammatory Pigmentation Model

For the UVB‐induced post‐inflammatory pigmentation model, mice from the same batch as those used for melasma modelling were randomly assigned to either a normal control group or a UVB treatment group. Within the UVB group, mice were further divided into head and ear modelling subgroups, with equal numbers in each. In the head modelling subgroup, a depilation area of approximately 1.5 x 1.5 cm^2^ was exposed to UVB radiation at 30 mJ/cm^2^ daily. For the ear modelling subgroup, UVB exposure was administered to the dorsal side of the left ear at a dose of 60 mJ/cm^2^, with the right ear and other regions protected. This modelling procedure was carried out over a 28‐day period, with mice anesthetized using mask‐delivered gas during each session. UVB radiation levels were monitored with a detector to ensure consistent exposure, and the effectiveness of tin foil shielding for non‐target areas was periodically tested.

### 2.4 Histopathological Sections and Index Detection

Following the modelling phase, skin samples from the targeted areas were collected. Tissue blocks were prepared for various analyses: sections were allocated for paraffin embedding, staining with H&E and Masson–Fontana and the preparation of tissue homogenates and supernatants, stored at −80°C. TYR levels were measured according to the instructions provided with the biochemical detection kit.

### 2.5 Pigmentation and Melanin Assessment

At the end of the experiment, mice were euthanized via inhalation of excess carbon dioxide. Initial visual inspections were conducted, followed by grey value analysis using ImageJ software. The head or ear skin from the modelling areas was carefully excised, fixed in 10% formaldehyde and embedded in paraffin for further examination. Skin sections were stained with haematoxylin and eosin (H&E) for general histology, and melanin was visualized using Masson–Fontana staining, examined under a microscope. Five random high‐power fields were selected from each melanocyte‐stained section, and ImageJ software v1.8 was used to automatically analyze staining (percentage of positive area), calculating the percentage of melanocytes in both the epidermis and dermis. The average of these measurements was recorded for analysis.

### 2.6 Detection of Serum Indicators

Upon completion of the modelling process, mice were prepared for serum collection by first removing the area around the whiskers. Gentle pressure was applied to the skin near the eyes to induce eyeball congestion and protrusion. Using elbow forceps, the eyeball was quickly clamped and removed, whilst gentle pressure was applied to the heart to collect blood into a sterile 1.5 mL centrifuge tube. Following blood collection, the mice were euthanized by cervical dislocation. The collected blood was left to clot at room temperature for 2 h, then centrifuged at 3000 rpm for 10 min to separate the serum. TYR, SOD and MDA levels were measured precisely according to the instructions provided with each biochemical kit.

### 2.7 Immunofluorescence Assay

Formalin‐fixed, paraffin‐embedded (FFPE) skin sections (5 μm thick) were subjected to immunofluorescence staining. Following antigen retrieval, sections were washed three times with PBS and blocked with 5% bovine serum albumin (BSA) containing 0.3% Triton X‐100 for 2 h at room temperature. They were then incubated overnight at 4°C with the primary antibody against LC3 (Abways, CY5992, 1:500 dilution)/p62 (PTG, 18,420‐1‐AP, 1:500 dilution). After rinsing, sections were incubated with Alexa Fluor 594‐conjugated secondary antibodies for 1 h at room temperature. Following three washes with Tris‐buffered saline containing Tween‐20 (TBST), nuclei were stained with 4′,6‐diamidino‐2‐phenylindole (DAPI) for 20 min. Fluorescence images were acquired using a laser scanning microscope (ECLIPSE, Nikon, Japan). Cultured cells were fixed with 4% paraformaldehyde for 15 min at room temperature, followed by three PBS washes. Cells were then blocked with 5% BSA containing 0.3% Triton X‐100 for 2 h and incubated overnight at 4°C with the LC3 primary antibody/p62 primary antibody. After washing with PBS, cells were incubated with Alexa Fluor 594–conjugated secondary antibodies/Dylight 488–conjugated secondary antibodies for 1 h at room temperature. Nuclei were counterstained with DAPI for 20 min. Images were captured using the same laser scanning microscope (ECLIPSE, Nikon, Japan).

### 2.8 Statistical Analysis

Statistical analysis of the experimental results was performed using GraphPad Prism 9 and SPSS 22.0 software. Comparisons between two groups were conducted using the independent sample t‐test, whilst one‐way ANOVA was used to analyze differences amongst multiple groups. A *p*‐value of less than 0.05 was considered statistically significant.

## 3 Results

### 3.1 Establishment and Validation of a Melasma Mouse Model

#### 3.1.1 Skin Appearance and Pigmentation Assessment in the Melasma Mouse Model

**FIGURE 1:**
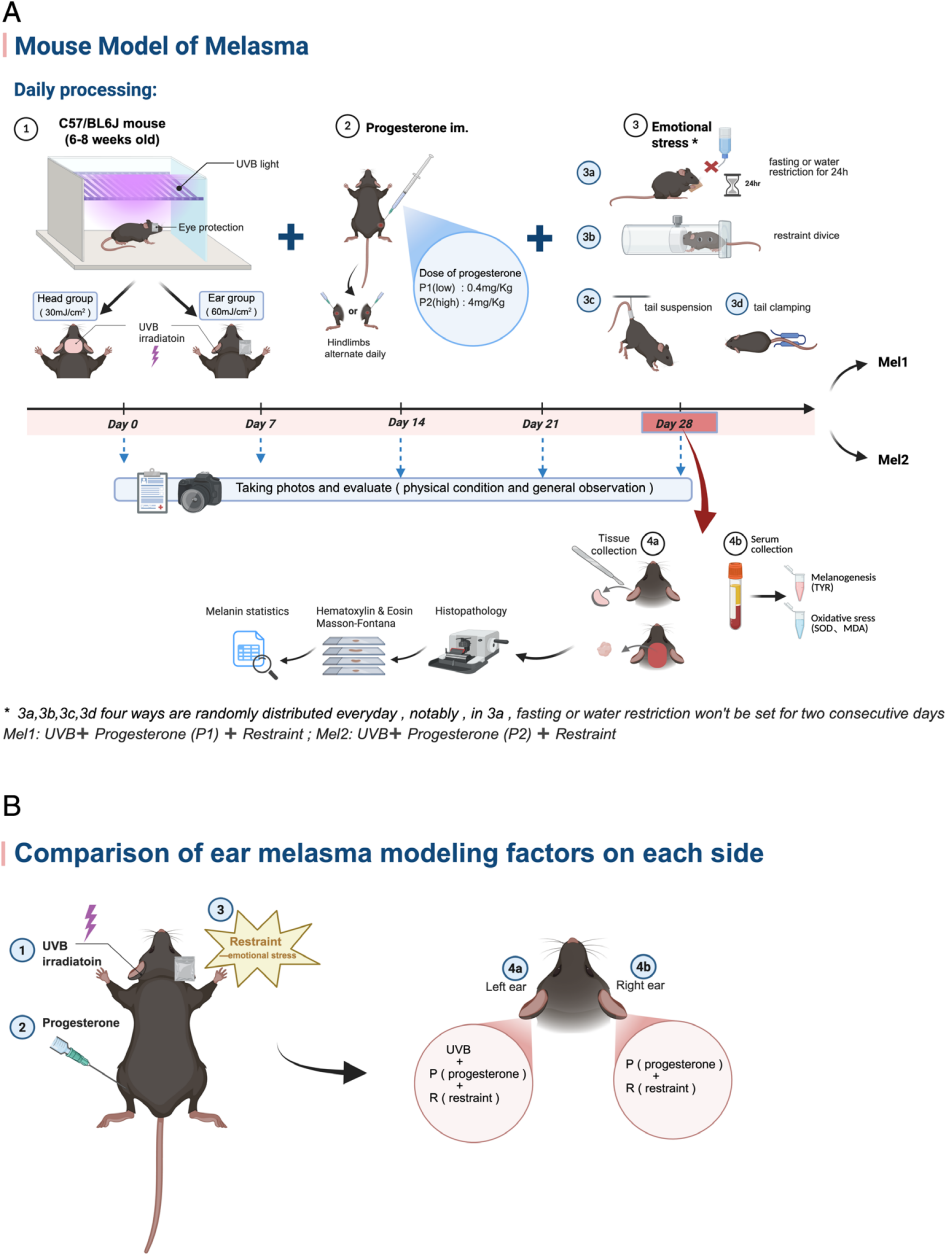
UVB irradiation combined with progesterone intramuscular injection and emotional stress (restraint) to establish a macular‐like mouse model. (A) ①: Broad spectrum UVB irradiates the shaved area of the mouse head (head group, 30 mJ/cm^2^) and the left ear of the mouse (ear group, 60 mJ/cm^2^, the right ear is covered by tinfoil), and tinfoil protects both eyes. ② Progesterone was injected intramuscularly (P1:0.4mg/kg in the low‐dose group, P2: 4mg/kg in the high‐dose group), and the two hindlimbs were injected alternately every day. ③ Emotional stress (restraint method). ③ A: Fasting or water deprivation for 24 h. ③ B: Restraint device for 5 min (the inner diameter of the restraint device is larger than the widest diameter of the mouse body, and there is a breathing hole). ③ C: Tail overhanging handstand for 2 min (the tail overhanging position will not over squeeze the tail of the mouse). The three restraint methods were applied on a rotating schedule and randomly assigned. Fasting or water deprivation was used only twice per week and never on consecutive days. The same order of arrangement is used during modelling between different groups. Three methods of joint modelling were carried out daily for 28 days. Photographs and observation records were taken every 7 days. After modelling, tissuesand serum (④ a) were collected for subsequent experiments. (B) The left ear (④ a) of the model mice was treated with UVB irradiation (①), progesterone (②) and chronic restraint factor (③), whilst the right ear (④ b) was only treated with progesterone factor (②) and chronic restraint factor (③) (created with BioRender.com).

**FIGURE 2:**
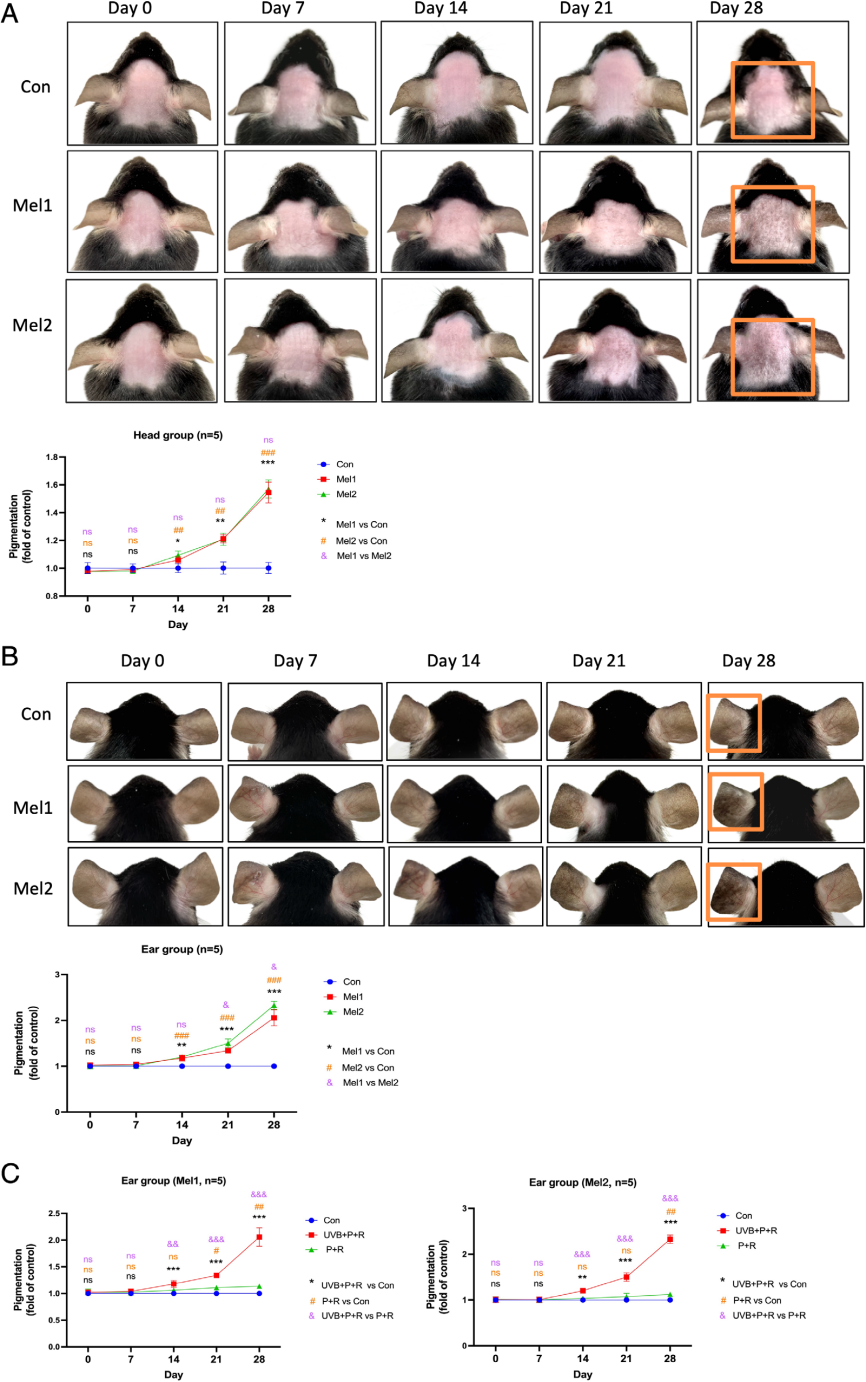
General observations of mice and evaluation of the pigmentation changes in the modelling area. (A) Evaluation of pigmentation in the shaved head area of melasma‐like and control mice, quantified using ImageJ software. Data are presented as mean ± SEM. */&/#: *p* < 0.05, **/&&/##: *p* < 0.01 and ***/&&&/###: *p* < 0.001. (B) Assessment of pigmentation in the shaved head area of melasma‐like and control mice, using the same method as in (A). */&/#: *p* < 0.05, **/&&/##: *p* < 0.01 and ***/&&&/###: *p* < 0.001. (C) Comparison of pigmentation in both ears of the melasma‐like mouse ear model group (different progesterone dose groups). */&/#:*p* < 0.05, **/&&/##: *p* < 0.01 and ***/&&&/###: *p* < 0.001.

We selected 6‐8‐week‐old female C57BL/6J mice and induced melasma over a 28‐day period using a combination of broad‐spectrum UVB, progesterone and restraint (Figure 1A). Macroscopic observations showed that, compared to controls, the melasma model mice (Mel1 and Mel2 groups) displayed mild erythema and desquamation on the head by day 7, without visible pigmentation (Figure 2A). By day 14, speckled pigmentation began to emerge, accompanied by slight desquamation. The pigmentation progressively darkened, merging into brown patches, and by day 28, the skin exhibited distinct irregular brown patches with central speckling, which closely resembled clinical melasma lesions. In the ear model group (Figure 2B), similar changes were observed. By day 7, the left UVB‐exposed ear in the model group showed mild wrinkling, desquamation and capillary dilation, with no visible changes in the right ear. Over time, the desquamation and vascular dilation resolved, and pigmentation gradually intensified in the affected areas. Overall skin observations suggest that C57BL/6J mice constitute an effective model for studying pigmented disorders such as melasma. Compared to ear modelling, head modelling yields a skin phenotype that more closely resembles clinical melasma.

We assessed the grayscale values of mouse skin using ImageJ software. At days 0 and 7, pigmentation levels in the head and ear model groups showed no significant difference from the control group. By day 14, however, pigmentation had significantly increased in the model groups compared to controls. Over the next 14 days, levels continued to rise, with the rate of intensification increasing through the end of the 28‐day period. No notable differences were observed in pigmentation trends between the two progesterone dose groups (Mel1 and Mel2) on the head. In the ear model group, however, Mel2 exhibited significantly greater pigmentation than Mel1 by day 21, which persisted until the end of the study. This may be due to the higher baseline melanin content in ear tissue, resulting in a more pronounced response to progesterone stimulation (Figure 2B). Further analysis of individual pigmentation trends for each mouse showed similar patterns (Figure S1).

In the ear model group, the left ear (UVB + *P* + *R*) was exposed to UV radiation, progesterone and stress, whilst the right ear (*P* + R) was influenced only by progesterone and stress (Figure 1B). To examine the roles of UV radiation, progesterone and stress in melasma modelling, we compared pigmentation levels between the left and right ears. In both the Mel1 and Mel2 groups, significant pigmentation appeared in the left ear (UVB + *P* + *R*) by day 14, with further intensification over time. In contrast, the right ear showed visible pigmentation only after day 21 or later; throughout the modelling period, the left ear consistently exhibited more pronounced pigmentation than the right (Figure 2C). Within‐group comparisons of individual mice showed similar pigmentation trends between the left and right ears (Figure S2). These results indicate that UV radiation plays a crucial role in melasma modelling, with progesterone and stress further aggravating pigmentation.

#### 3.1.2 Enhanced Melanin Deposition and Synthesis in the Melasma‐Like Mouse Model

**FIGURE 3:**
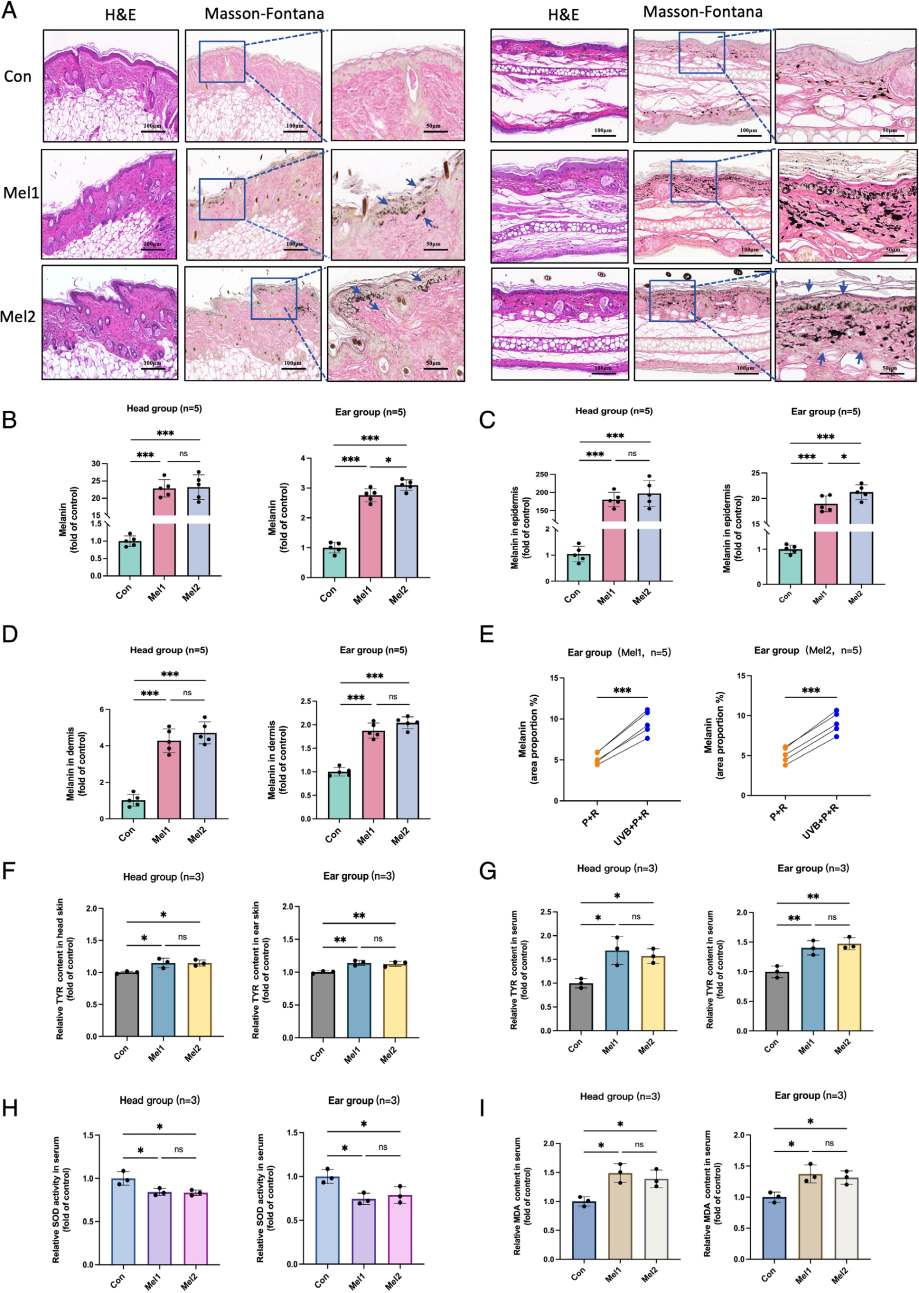
Histopathological staining, melanin quantification, TYR, SOD and MDA level assessments in mice. (A) H&E staining and Masson–Fontana melanin staining of the modelled and control areas, with enlarged views. The left panel shows the head model region, and the right panel shows the ear model region. (B) Ratio of melanin granules to total epidermal and dermal area in the local skin of mice. *p < 0.05, **p < 0.01 and ***p < 0.001. (C) Ratio of melanin granules to epidermal area in mouse skin. *p < 0.05, **p < 0.01 and ***p < 0.001. (D) Ratio of melanin granules to dermal area in mouse skin. *p < 0.05, **p < 0.01 and ***p < 0.001. (E) Comparison of melanin content in both ears of the ear model group (different progesterone doses), with the left ear treated with UVB + *P* + *R* and the right ear treated with *P* + R. *p < 0.05 and ***p < 0.001.(F, G) TYR content in local skin and serum of mice. *p < 0.05 and **p < 0.01.(H, I) SOD activity and MDA content in mouse serum. *p < 0.05 and **p < 0.01.

After the modelling period, skin tissues were analyzed using H&E and Masson–Fontana melanin staining. H&E staining revealed features of solar elastosis in both head and ear melasma groups, including damage to the stratum corneum, mild epidermal thickening, disorganized collagen in the superficial dermis and melanin granule deposition in both the epidermis and dermis. Melanin staining showed that, in the control group, only sparse melanin granules were observed in the dermis and around hair follicles in the head area. In contrast, melasma‐like mice exhibited a significant increase in melanin granules throughout the full epidermal layer, along with scattered melanin granules and melanophages in the dermis. Abundant melanin granules were present in the basal, spinous and corneal layers in a band‐like pattern, with occasional melanin caps in the basal layer (Figure 3A). In the ear control group, both the dorsal and ventral sides of normal skin showed minimal, scattered melanin granules in the dermis, with similar levels on each side. However, in the left (UVB‐exposed) ear of melasma‐like mice, melanin was deposited throughout the epidermis, with a continuous distribution in the basal layer and some melanin extending into the dermis, likely transported by downward‐projecting melanocytes. The dermis showed a marked increase in melanin granules and melanophages, closely resembling the pathology of clinical melasma. Additionally, the ventral (unexposed) side of the left ear in the melasma‐like mice displayed a significant reduction in dermal melanin compared to controls.

Quantification of melanin granules using ImageJ software revealed that the proportion of melanin granules in both the epidermis and dermis of the head and ear skin was significantly higher in the melasma model groups compared to controls, with no notable difference between the Mel1 and Mel2 groups (Figure 3B). Separate analyses of the epidermis and dermis showed markedly elevated melanin levels in the head skin of the melasma model groups relative to controls, again with no significant variation between Mel1 and Mel2. In the ear model skin, both epidermal and dermal melanin levels were significantly higher than those in controls, with melanin deposition being more pronounced in Mel2 than in Mel1 (Figure 3C,D). A comparison of melanin deposition between the left (UVB + *P* + *R*) and right (P + R) ears further indicated that pigmentation was significantly greater in the left ear (Figure 3E). These results suggest enhanced epidermal and dermal melanin deposition in the melasma‐like mouse model, with UV radiation as a key factor in melasma induction, further amplified by progesterone and stress. Additionally, both local and serum TYR levels were significantly elevated in the melasma model groups compared to controls, with no significant difference between Mel1 and Mel2, suggesting an increased melanin synthesis capacity in the melasma‐like mice independent of progesterone dosage (Figure 3F,G).

#### 3.1.3 Elevated Oxidative Stress Levels in the Melasma‐Like Mouse Model

Beyond the formation of facial brown patches, melasma is often accompanied by systemic changes, such as oxidative stress imbalance, which is a known contributor to its pathogenesis [26]. SOD is a critical antioxidant enzyme that neutralizes superoxide anion radicals, thereby protecting cells from oxidative damage [27]. MDA, a product of lipid peroxidation due to ROS, serves as a reliable indicator of oxidative stress [28, 29]. Following melasma modelling, SOD levels in both the ear and head model groups were significantly lower than those in controls, whilst MDA levels were significantly higher, with no notable difference between the Mel1 and Mel2 groups (Figure 3H,I). These results indicate elevated oxidative stress levels in the melasma‐like mouse model.

Considering the clinical phenotype, histopathological features, and changes in related indicators, both progesterone model groups (Mel1 and Mel2) successfully replicated the lesion characteristics seen in clinical melasma patients, along with similar pathological changes associated with the condition. Given the lack of significant differences between the Mel1 and Mel2 groups, and in adherence to animal ethics, the low‐dose progesterone group (Mel1) was chosen as the appropriate modelling dosage.

### 3.2 Comparison Between the Melasma Model and the Post‐Inflammatory Hyperpigmentation Mouse Model

#### 3.2.1 Distinctions Between the Melasma‐Like Mouse Model and the UVB‐Induced Hyperpigmentation Model

**FIGURE 4:**
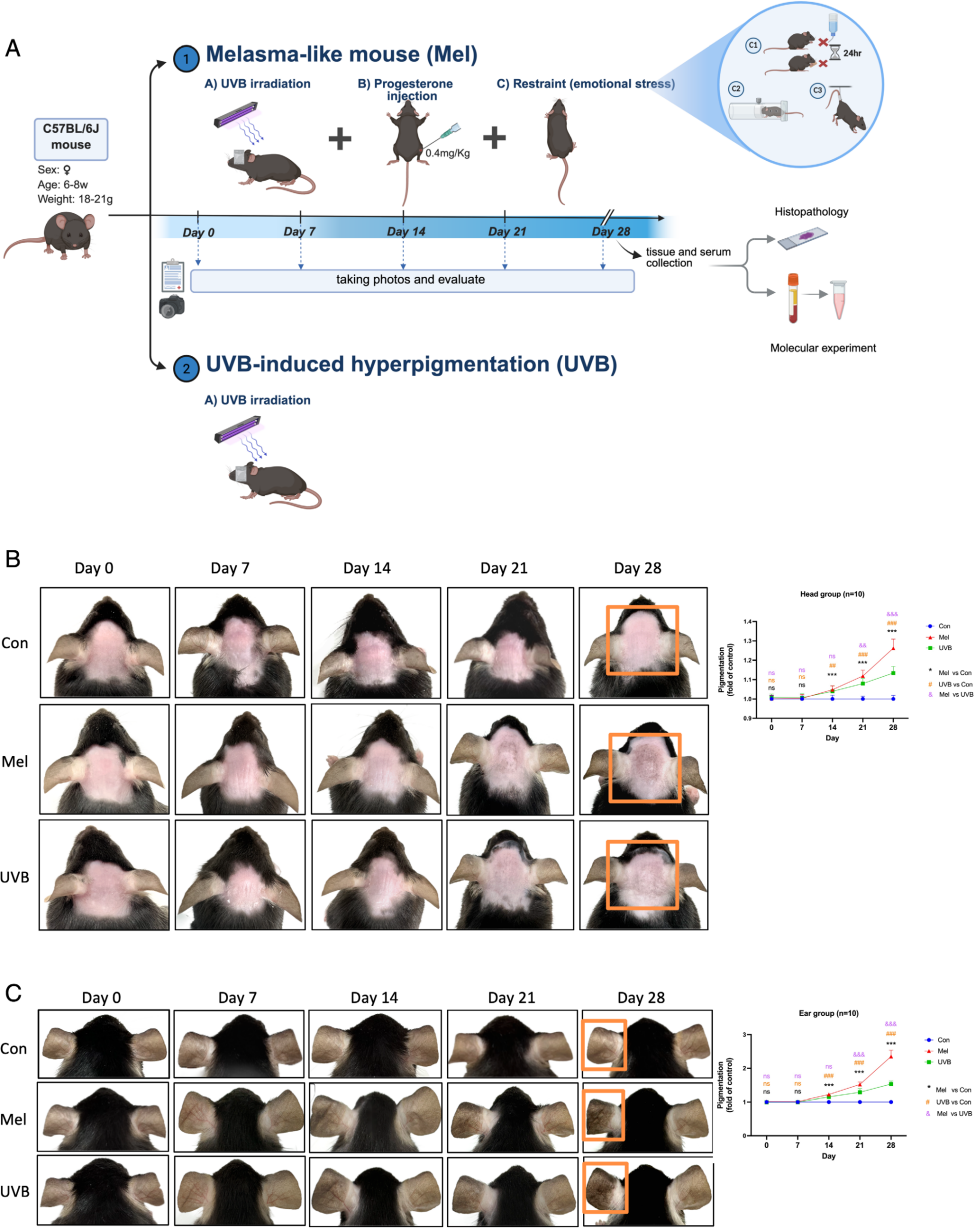
Comparison between the mouse model of increased pigmentation induced by UVB irradiation alone and the melasma‐like mouse model induced by three factors. (A) The melasma‐like mouse model was established as described in Section 1, with a low‐dose progesterone injection (0.4mg/kg). The UVB‐induced hyperpigmentation model was created using the same UVB dose as in the melasma model, with daily exposure for 28 days. Photographs and observations were recorded every 7 days, and tissues and serum were collected post‐modelling for further experiments (created with BioRender.com). (B, C) Pigmentation Assessment:Pigmentation levels were quantified using the same method as in Figure 2. Data are presented as mean ± SEM. */#/&: *p* < 0.05, **/##/&&: *p* < 0.01 and ***/###/&&&: *p* < 0.001.

In comparing the melasma‐like mouse model (Mel) with the UVB‐induced hyperpigmentation model, we observed that both the Mel and UVB groups exhibited mild erythema and desquamation in the irradiated skin area by day 7. By day 14, erythema and desquamation had diminished, giving way to visible pigmentation. Pigmentation continued to deepen, and by day 28, the Mel group's head displayed irregular brown patches with centrally speckled dark brown pigmentation, while the UVB group's head showed lighter brown patches. Pigmentation was more pronounced in the Mel group than in the UVB group (Figure 4B), with similar patterns observed in the ear model (Figure 4C). Grayscale analysis further revealed that, compared to controls, pigmentation in both the head and ear areas of the Mel and UVB groups increased by day 7, with no significant difference between the Mel and UVB groups at this point. However, pigmentation continued to intensify in both groups, and after day 14, the Mel group exhibited significantly more pronounced pigmentation than the UVB group (Figure 4B,C).

General skin observations and grayscale analysis revealed that pigmentation in the Mel group—induced by a combination of UV irradiation, intramuscular progesterone and stress—was significantly more pronounced than in the UVB‐only group. The Mel group's head skin exhibited brown patches with speckled pigmentation, closely resembling clinical melasma, whereas the UVB group displayed lighter brown patches on the head.

#### 3.2.2 Histopathological Evaluation

**FIGURE 5:**
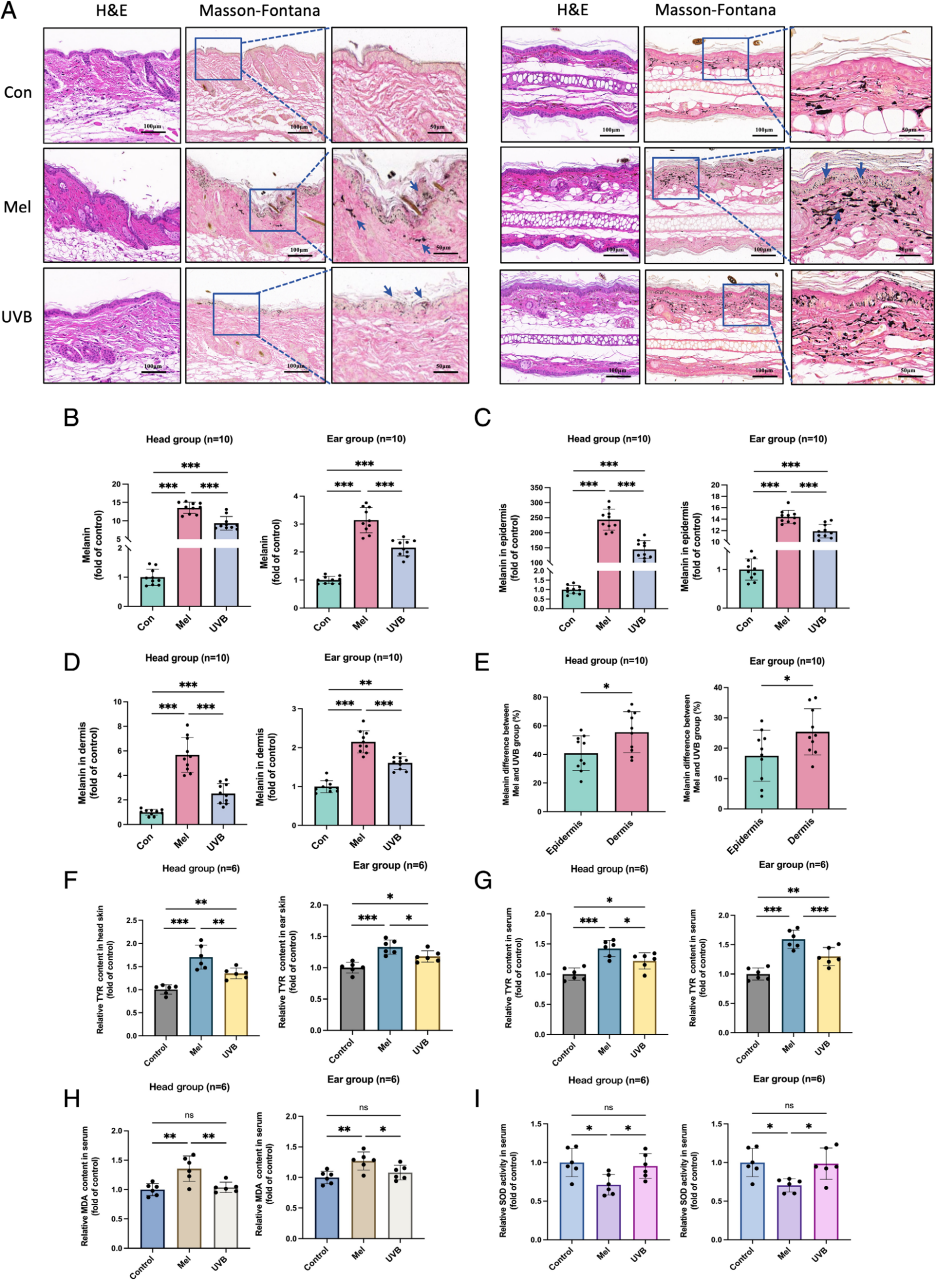
Histopathological staining of local skin, quantitative evaluation of melanin particles, indicators of melanin synthesis and detection of oxidative stress levels in vivo for mice. (A) Pathological H&E staining and Masson–Fontana melanin staining, along with magnified images of the modelling and control areas; the left image shows the head modelling area, whilst the right shows the ear modelling area. (B–D) Quantitative assessment of melanin in mouse skin, calculated as per the method in Figure 3. (E) Comparison of epidermal and dermal melanin granule increases between the melasma and UVB groups, with significance levels indicated as *p* < 0.05, **p < 0.01 and ***p < 0.001. (F, G) Measurement of TYR levels in local skin and serum of mice. (H, I) Assessment of serum SOD activity and MDA levels in mice.

H&E staining revealed that both the Mel and UVB groups exhibited characteristics of solar elastosis and melanin deposition in the epidermis and dermis of the head and ear. Masson–Fontana melanin staining showed a significant increase in melanin across the entire epidermal layer of the head in melasma‐like mice, with abundant melanin granules in the basal layer and a marked rise in scattered melanin granules and melanophages in the dermis. In contrast, the UVB group showed a primarily epidermal melanin increase in the head, with a discontinuous distribution of melanin granules in the basal layer, less pronounced than in the Mel group and minimal melanin deposition in the dermis. In the left (UVB‐exposed) ear of the Mel group, dense melanin granule deposition was observed across the full epidermal layer, with a continuous distribution of melanin in the basal layer and significant melanin accumulation in the dermis. In the UVB group's left ear, melanin accumulation was primarily confined to the basal layer of the epidermis, with a discontinuous distribution of melanin granules and a moderate increase in dermal melanin, though less pronounced than in the Mel group (Figure 5A).

The assessment of melanin granules using Image J software reveals that both the Mel and UVB groups exhibit significantly higher total melanin granules in the epidermis and dermis compared to the control group, with the Mel group exhibiting a more substantial increase than the UVB group (Figure 5B). Melanin granule levels are elevated in both the epidermis and dermis of the Mel and UVB groups relative to the control, with the Mel group showing a markedly greater increase than the UVB group in both areas (Figure 5C,D). Additionally, the increase in dermal melanin is especially prominent in the Mel group (Figure 5E). These findings indicate that both melasma modelling and post‐inflammatory hyperpigmentation lead to melanin deposition in the epidermis and dermis, with more pronounced melanin accumulation observed in the Mel group, particularly within the dermis.

The TYR assay results further show elevated TYR levels in the skin and plasma of the Mel and UVB groups compared to the control, with the Mel group exhibiting a significantly higher increase than the UVB group (Figure 5F,G). This suggests that the Mel group has a greater melanin synthesis capacity than the UVB group.

An additional interesting phenomenon was observed in the Mel and UVB groups: on the non‐irradiated (shadowed) side of the left ear, dermal melanin was significantly reduced compared to the control, whilst epidermal melanin showed no notable difference (Figure S2B–D). In both groups, the total epidermal melanin on the irradiated and shadowed sides of the left ear was substantially higher than in the control (Figure S2E–G), whereas total dermal melanin showed no significant difference from the control (Figure S2H–J).

#### 3.2.3 Oxidative Stress Levels In Vivo

UV radiation significantly influences oxidative stress in both the skin and the body. UV exposure triggers the production of large quantities of ROS and free radicals through interactions with skin pigments, such as melanin and lipids. This process damages cell membranes, proteins and DNA, leading to oxidative stress [30, 31]. Short‐term, low‐dose UV exposure generally limits free radicals and inflammatory mediators to the skin, whereas prolonged, high‐dose exposure can lead to systemic oxidative stress and broader health complications. Melasma is frequently associated with a systemic oxidative stress response. Our findings reveal that during modeling, serum MDA levels in the Mel group were significantly higher than those in both the control and UVB groups, whilst the UVB group showed a slight, non‐significant increase compared to the control. Serum SOD levels in the Mel group were also substantially elevated relative to both the control and UVB groups, with no difference between the UVB and control groups (Figure 5H,I). These results suggest that in melasma, factors beyond UV exposure—including complex interactions and alterations in the microenvironment—contribute to elevated systemic oxidative stress levels.

### 3.3 Tranexamic Acid Intervention in a Melasma Mouse Model

#### 3.3.1 Intervention Model Method With Tranexamic Acid

**FIGURE 6:**
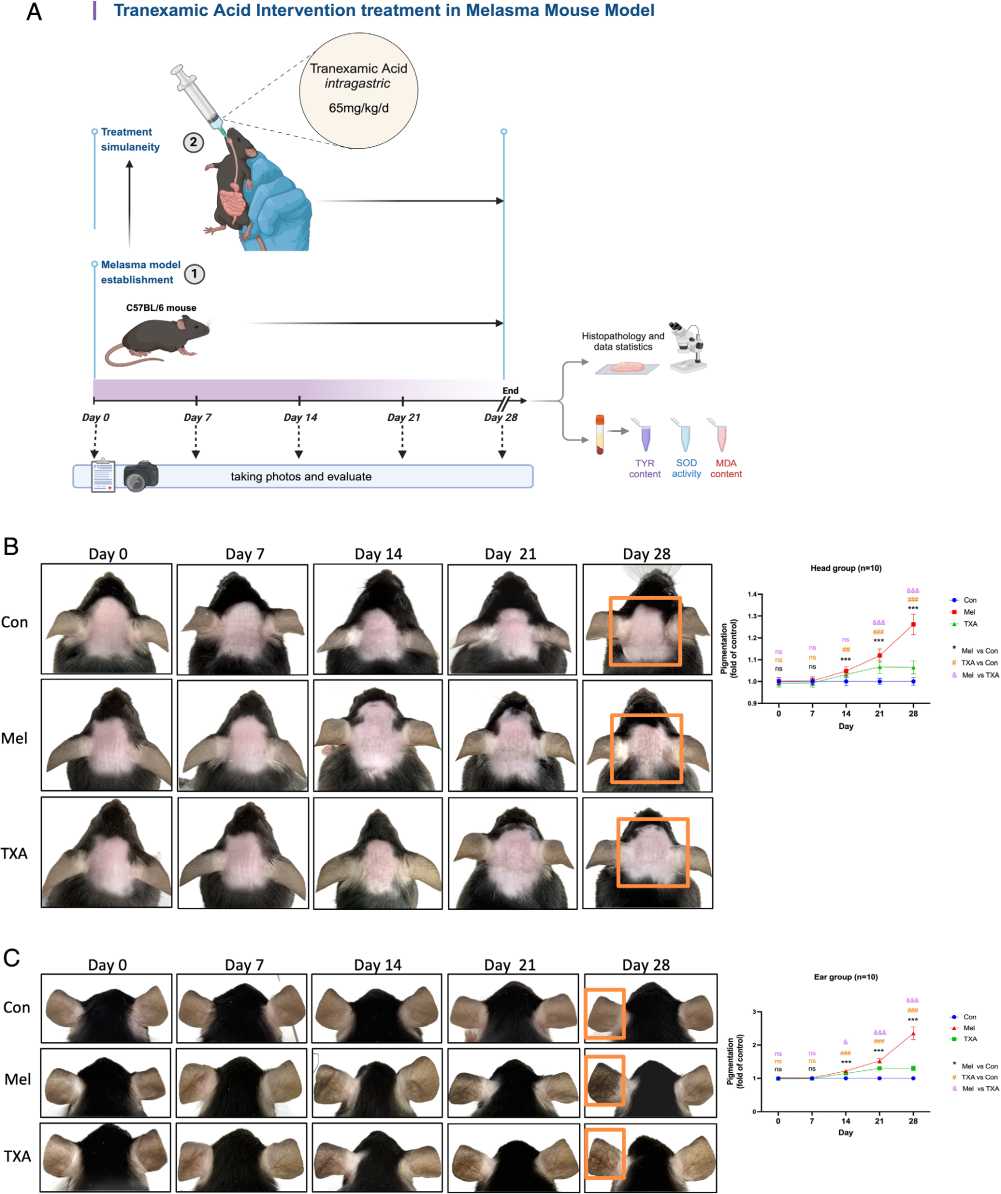
Effect of tranexamic acid gavage on the degree of pigmentation in melasma‐like mice. (A) During melasma modeling, mice were administered a daily oral gavage of tranexamic acid at a dose of 65 mg/kg for 28 days. Observation and recording methods were consistent with previous procedures described (figure created with BioRender.com). (B, C) Documentation of pigmentation levels in melasma‐like mice, the tranexamic acid treatment group and the control group, with calculations following the method in Figure 2. Data are presented as mean ± SEM. */#/&: *p* < 0.05, **/##/&&: *p* < 0.01 and ***/###/&&&: *p* < 0.001.

Tranexamic acid, a widely used anticoagulant, inhibits fibrinolysis by blocking plasmin activity. In the body, it also suppresses TYR activity, disrupting melanin and keratinocyte formation and helping alleviate symptoms in melasma patients. To further investigate its effects on melasma, we administered a daily oral gavage of tranexamic acid (65 mg/kg, equivalent to the standard treatment dose for melasma patients) to age‐ and sex‐matched C57BL/6J mice during melasma modelling. This group, referred to as the TXA group, was compared with the control and Mel groups (Figure 6A).

#### 3.3.2 Phenotypic Observations

On day 7, both the Mel and TXA groups showed mild erythema and scaling in the skin of the head area compared to the control group, without visible pigmentation. By day 14, spotty pigmentation appeared on the heads of both groups. Over the following days, pigmentation in the Mel group continued to deepen until the end of the 28‐day modelling period, whilst in the TXA group, it began to gradually lighten after day 21, resulting in significantly reduced pigmentation compared to the Mel group by day 28. A similar pattern was observed in the ear modelling area (Figure 6B,C).

Grey scale analysis showed that pigmentation in the head and ear areas of the TXA group began to lighten compared to the Mel group by day 14, with more noticeable reduction after day 21 (Figure 6D). These results suggest that tranexamic acid can mitigate pigmentation in melasma‐like mice, with observable effects beginning around days 14 to 21.

#### 3.3.3 Pathology and Melanin Content Analysis

**FIGURE 7:**
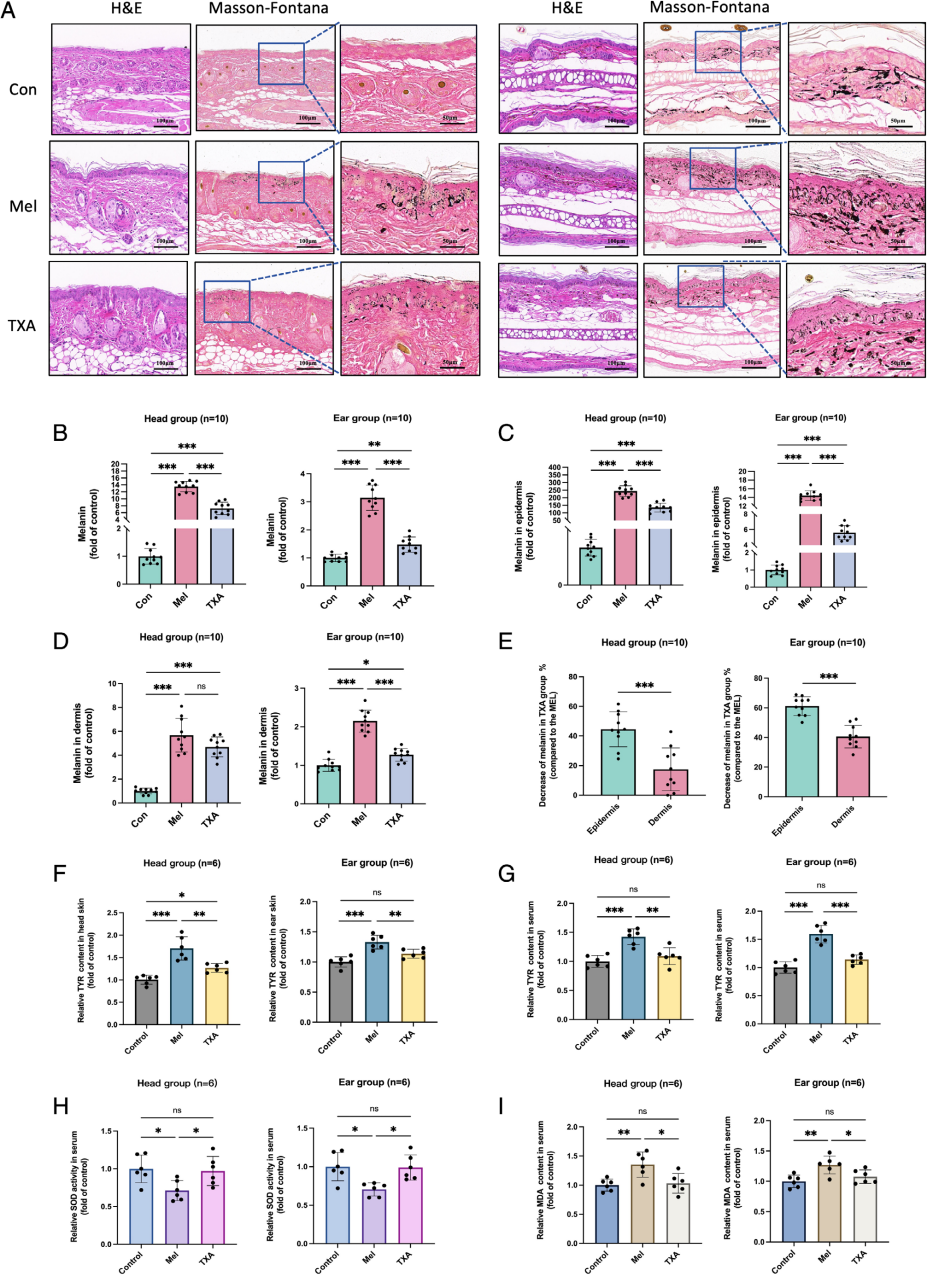
Histopathological staining of local skin, quantitative assessment of melanin particles, evaluation of melanin synthesis indicators and in vivo detection of oxidative stress levels in mice. (A) Pathological H&E staining and Masson–Fontana melanin staining with magnified images of the modelling and control areas; the left image shows the head modelling area, whilst the right shows the ear modelling area. (B–D) Quantitative evaluation of melanin in mouse skin, calculated as described in Figure 3. (E) Comparison of epidermal and dermal melanin granule increases between the melasma and tranexamic acid treatment groups, with significance levels indicated as *p < 0.05, **p < 0.01 and ***p < 0.001. (F, G) Measurement of TYR levels in local skin and serum of mice. (H, I) Assessment of serum SOD activity and MDA levels in mice.

H&E staining revealed solar elastosis and melanin deposition in the superficial dermis in both the Mel and TXA groups compared to the control. Melanin staining showed that whilst melanin deposition in the superficial dermis was more pronounced in the TXA group than in the control, it was significantly reduced compared to the Mel group (Figure 7A). Quantitative analysis of melanin granule distribution indicated increased melanin in the superficial dermis, epidermis and dermis individually in both the Mel and TXA groups compared to the control, with the TXA group showing a marked reduction relative to the Mel group (Figure 7B–D). Additionally, the reduction of melanin in the epidermis was more substantial in the TXA group than in the Mel group (Figure 7E), suggesting that tranexamic acid alleviates pigmentation in melasma‐like mice primarily by reducing melanin deposition in the epidermis.

TYR assay results showed elevated TYR levels in the head and ear modelling areas in both the TXA and Mel groups compared to the control, though levels were significantly lower in the TXA group than in the Mel group. Additionally, serum TYR levels in the TXA group were significantly reduced compared to the Mel group and were similar to those in the control (Figure 7F,G). These findings suggest that oral administration of tranexamic acid reduces TYR levels in the skin and serum of melasma‐like mice, thereby decreasing melanin synthesis and alleviating pigmentation.

#### 3.3.4 Oxidative Stress Levels

In addition to inhibiting melanin synthesis, tranexamic acid may also alleviate melasma by reducing oxidative stress‐related tissue damage [32]. Our results show that serum SOD activity in the TXA group was significantly higher than in the Mel group, while MDA levels were reduced to levels comparable to the control group (Figure 7H,I). These findings suggest that tranexamic acid can reverse oxidative stress abnormalities in melasma‐like mice, restoring them closer to normal levels.

### 3.4 Altered Autophagy Levels in Melasma Model Mice

**FIGURE 8:**
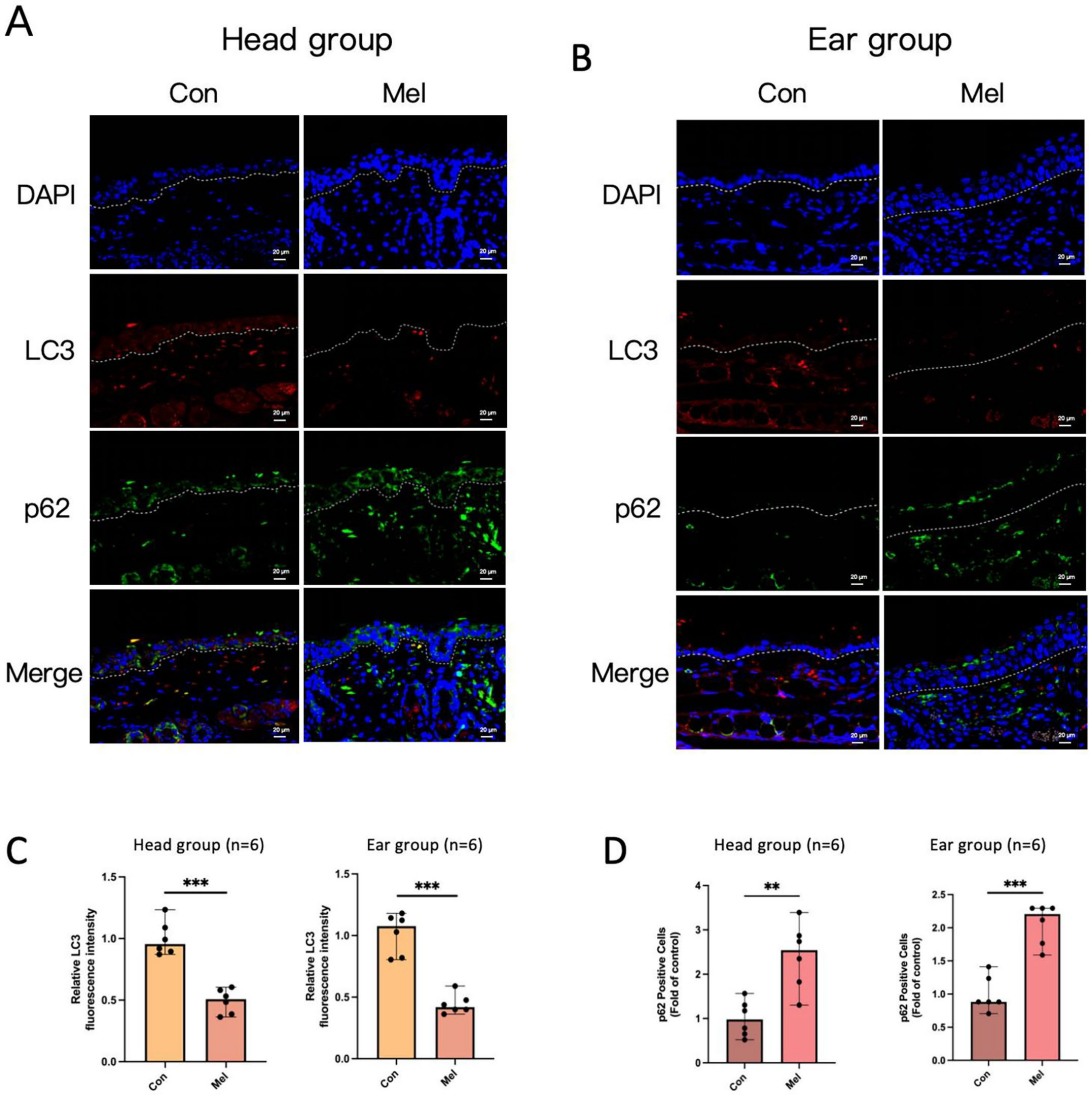
Immunofluorescence staining for the detection of autophagy‐related proteins in mouse skin. (A) Immunofluorescent analysis demonstrated fluorescence staining of LC3 expression (red) and p62 expression (green) in mouse head skin. (B) Immunofluorescent analysis demonstrated fluorescence staining of LC3 expression (red) and p62 expression (green) in mouse ear skin. Merged images with DAPI nuclear staining were shown. Bars = 20 μm. (C, D) The relative fluorescence intensities of LC3 and p62 were quantified utilizing ImageJ software. **p < 0.01 and ***p < 0.001.

Previous studies have shown that autophagy is frequently downregulated in melanocytes, keratinocytes and dermal fibroblasts within melasma lesions [21, 22, 23, 24]. Immunofluorescence staining of autophagy‐related proteins in this study revealed that the expression level of LC3, a key marker of autophagy, was decreased in the skin lesions of mice in the Mel group compared to the Con group. In contrast, the expression level of p62, an autophagy receptor that accumulates when autophagy is impaired, was significantly increased in the skin lesions of mice in the Mel group compared to the Con group (Figure 8A–D). These findings indicate a reduction in autophagic activity in the lesional skin of melasma‐like model mice.

## 4 Discussion

Melasma is a persistent and treatment‐resistant pigmentation disorder, often worsened by factors such as UV exposure, environmental influences and hormonal fluctuations [33]. Developing a suitable animal model is crucial for exploring its molecular mechanisms and potential prevention strategies. This study aims to establish a stable and reliable animal model for melasma to support future research efforts.

Effective animal models for melasma are currently lacking. In recent years, attempts have been made to induce melasma using methods such as UV exposure, progesterone administration and restraint [34]. However, these approaches suffer from low reproducibility and poor stability, often lacking essential details on animal strains, UV dosage, exposure methods and phenotype assessment data. Although comparative studies involving different animal strains, sexes and ages have been conducted [35, 36], they still lack detailed protocols, parameters and phenotype validation. Thus, an effective animal model that accurately reflects the clinical characteristics of melasma has yet to be established.

To establish a reliable modeling method, we developed a melasma mouse model using a combination of UV exposure, intramuscular progesterone and emotional stress over a 28‐day period. Preliminary trials were conducted with commonly used laboratory mice, including C57BL/6J, Balb/c and KM strains, with C57BL/6J ultimately identified as the most suitable for further modelling. In this study, we systematically screened and validated each factor in the modelling process to determine its relative importance. We tested various UV doses to identify the minimum exposure levels needed to produce clinically relevant phenotypes, selecting optimal doses of 30 mJ/cm^2^ for the head and 60 mJ/cm^2^ for the ears. Two progesterone doses (0.4 mg/kg and 4 mg/kg) were also evaluated, with no significant differences in model phenotype between the doses. This suggests that whilst progesterone may not be essential, it likely interacts synergistically with UV exposure to produce the desired phenotype. Additionally, we compared the effects of a three‐factor combination (UVB + progesterone + restraint) with a two‐factor model (progesterone + restraint) on skin changes associated with melasma. Results indicated that UV‐exposed mice displayed significantly more pronounced pigmentation and skin changes that closely resembled clinical melasma, with clear differences from the non‐UV model. These findings underscore the essential role of UV exposure in creating an effective melasma model. With the combined application of all three factors, the C57BL/6J mice demonstrated skin responses in the modelled areas consistent with clinical features of melasma, including brown pigmented patches, increased melanin in the epidermis and dermis, enhanced local and systemic melanin synthesis and elevated systemic oxidative stress levels. These results confirm that we successfully established an effective and reliable melasma mouse model suitable for experimental research.

UVB radiation is a central factor in melanin production and a primary trigger for melasma [37, 38]. UVB can stimulate melanin synthesis directly and indirectly by influencing keratinocytes, mast cells and fibroblasts [39, 40]. To exclude a simple UV‐induced tanning response, we developed a UVB‐induced pigmentation model. Results revealed that dermal melanin deposition was significantly higher in the Mel group compared to the UVB group, primarily due to an increase in dermal melanin granules. This finding suggests that in melasma, skin barrier dysfunction and basement membrane disruption are more prevalent, allowing basal layer melanocytes to produce melanin upon UV exposure, which then fall into the dermis, leading to a significant presence of dermal melanin granules and melanophages [41, 42, 43, 44]. Additionally, the Mel group showed a marked increase in TYR levels and systemic oxidative stress, indicating that factors beyond UV exposure—such as progesterone and emotional stress—contribute to systemic microenvironmental changes in the model [45, 46]. Therefore, our melasma model is not solely UV‐induced; progesterone and emotional stress play crucial roles, suggesting that UV exposure alone may be insufficient to fully replicate the complexity of melasma.

We further investigated the effects of tranexamic acid on the melasma model. Several studies indicate that oral tranexamic acid is more effective than topical application as a first‐line treatment for melasma [47]. Tranexamic acid acts by inhibiting plasmin activity, reducing the release of melanocyte‐stimulating mediators, suppressing melanin synthesis, decreasing mast cell activity and reducing the number of dermal blood vessels and mast cells. It also offers anti‐photoaging benefits and helps regulate oxidative stress [48, 49, 50, 51]. Our study found that gavage‐administered tranexamic acid not only reduced skin pigmentation but also corrected systemic oxidative stress imbalance, suggesting that its effects may be systemic. This aligns with clinical observations that oral administration is more effective than topical application, providing a valuable model for further research on tranexamic acid's mechanisms and optimal administration methods.

To further assess the specificity of the melasma‐like mouse model, we investigated potential underlying mechanisms. The pathogenesis of melasma is known to be complex, involving multiple genes and signalling pathways; however, suitable biomarkers for accurate diagnosis and characterization remain elusive. A key pathogenic feature of melasma is the hyperactivity of melanocytes, rather than an increase in melanocyte number. Several studies have suggested that impaired autophagy plays a critical role in the persistent melanogenesis observed in melasma [23, 24, 25]. In our study, immunofluorescence staining of skin sections from melasma‐like mice revealed reduced local LC3 levels and increased p62 expression compared to controls, indicating a decrease in autophagic activity. These findings align with previous reports and suggest that pigmentation in the melasma‐like model is not solely attributable to UV‐induced tanning, but rather may reflect altered autophagy within local skin cells. This autophagic dysfunction likely contributes to microenvironmental changes that promote melanogenic hyperactivity and impaired melanosome degradation. Taken together, these results indicate that the melasma‐like mouse model closely mirrors the pathophysiological features of clinical melasma. Furthermore, targeting and enhancing autophagy in melanocytes and associated skin cells may offer a promising therapeutic strategy for treating melasma.

Interestingly, in the melasma mouse model with the ear as the modelling site, we observed that UV‐induced pigmentation resulted in significantly increased melanin in the superficial dermis on the UV‐exposed (irradiated) side of the ear, while dermal melanin on the non‐UV‐exposed (shadowed) side was notably reduced compared to the control, with no significant difference in epidermal melanin. This phenomenon appeared consistently across all modelling batches. Further analysis of melanin content revealed that total epidermal melanin on both the irradiated and shadowed sides was significantly higher than in the control, whilst total dermal melanin levels remained close to normal. This suggests that the increase in epidermal melanin on the irradiated side results from UVB‐activated melanocytes, whilst dermal melanin may transfer from the shadowed side. Notably, previous studies in which the irradiated side was switched by shielding it and exposing the opposite side showed similar results. We speculate that this may be due to increased melanin synthesis on the irradiated side, potentially depleting the precursors and proteases needed for melanin production, or that a unique communication system among melanocytes maintains relative dermal melanin balance across both sides of the ear cartilage. Although further investigation into these mechanisms was beyond the scope of this study, future studies could involve fluorescent labelling of melanin granules and skin sectioning at multiple time points during modelling to explore these possible mechanisms.

This study has certain limitations. First, in our melasma modelling approach, we selected progesterone injections instead of oestrogen. Oestrogen has a broad range of effects, and administering high doses of exogenous oestrogen could introduce risks such as mammary hyperplasia, endometrial disorders and tumours in mice. For ethical and humane considerations, we chose the milder effects of progesterone. However, as oestrogen plays a significant role in melasma, further research is needed to determine an appropriate oestrogen dosage for melasma modelling that minimizes adverse side effects in mice. Comparing different modelling approaches will also help identify methods that better replicate the clinical features of melasma. Second, this study used UVB irradiation for modelling. Recent research, however, suggests that UVA and visible light also contribute to melasma development [52]. In future studies, we plan to use a solar simulator to replicate a composite light source, closely resembling the actual environmental exposures of melasma patients. Additionally, the role of restraint‐induced emotional stress was not investigated independently, leaving the specific impact of restraint on melasma modelling unclear. Future research will evaluate the application value of this factor. Lastly, we assessed TYR, a marker of melanin synthesis and oxidative stress indicators SOD and MDA, validating the local autophagy markers such as LC3 and p62, without exploring newer or potentially more effective indicators for evaluating melasma. In future studies, we may conduct transcriptomics or single‐cell sequencing on melasma‐like mice to identify differentially expressed molecules in melasma. These findings will be validated in animal models and melasma patients to discover relevant biomarkers that can guide diagnosis and treatment.

## 5 Conclusion

In this study, we developed a C57BL/6J mouse model of melasma using a combination of UV radiation, progesterone and emotional stress and evaluated it through morphological and biological assessments. We compared this model with a UV‐induced post‐inflammatory hyperpigmentation model and examined the effects of tranexamic acid on melasma‐like mice. This comprehensive approach validated a stable and clinically relevant animal model for melasma, providing a scientific foundation for the establishment and selection of melasma models.

## Author Contributions

T.L., X.C. and H.X. designed, supervised and directed the study; W.W. wrote the original draft; W.W., X.S. and Y.L. conducted experiments and analyzed data. T.L., X.C., H.X., Y.L. and H.Y. revised the draft. Y.Y., X.Z. and X.L. conducted experiments. * W.W., X.S. and Y.L. should be considered joint first authors. % T.L., X.C. and H.X. should be considered corresponding authors.

## Conflicts of Interest

The authors declare no conflicts of interest.

## Supporting information


**Data S1.** Supplementary Figure.

**Data S2.** Supporting Information.

## Data Availability

Data sharing is not applicable to this article as no new data were created or analyzed in this study.
