# Supplementary material for: Establishment and Validation of a C57BL/6J Mouse Model for Melasma
Source: Cell Prolif. 2025 Jul 10;59(1):e70078. doi: 10.1111/cpr.70078 (PMC12774621; doi:10.1111/cpr.70078)
Supplement: Supplementary file 1 — Data S1. Supplementary Figure. [file CPR-59-e70078-s001.zip › Figures S1 and S2_Captions.pdf]

Fig. S1. Statistical curve of gray value representing mouse color shade. (A) Two distinct progesterone doses were administered to establish the melasma-like mouse group and to assess the shade contrast with the control group. (B) Comparison of pigmentation levels between the left ear (influenced by three factors: UVB+P+R) and the right ear (affected by two factors: P+R) of melasma-like mice and the control group. (C) Analysis of pigmentation degrees among the melasma-like mouse group, UVB-induced pigmentation mice, and the control group. (D) Evaluation of color precipitation in the tranexamic acid intervention group, melasma-like mouse group, and control group. The calculation methodology follows that outlined in Fig. 2. Data are presented as mean ± SEM. Statistical significance is denoted as follows: */#/& $P < 0.05$, **/\\/&& $P < 0.01$, ***/\\\&&& $P < 0.001$. (Comparison of differences within groups).

Fig. S2. Melanin particle statistics in the epidermis and dermis of the irradiated and shaded sides of the ears in melasma-like mice and control mice. (A) Microscopic schematic diagram of the ears of mice with melasma receiving UV radiation. The dorsal side of the ears exposed to UV radiation is defined as the irradiated side, while the ventral side not exposed to UV radiation is defined as the shaded side. (B-D) Melanin particle statistics in the ventral (shaded side) epidermis (left) and dermis (right) of ears across different mouse groups. (E-G) Epidermal melanin particle statistics on both sides of the ears in various mouse groups. (H-J) Dermal melanocyte statistics on both sides of the ears in different mouse groups.
