# Supplementary figures and images for: Establishment and Validation of a C57BL/6J Mouse Model for Melasma
Source: Cell Prolif. 2025 Jul 10;59(1):e70078. doi: 10.1111/cpr.70078 (PMC12774621; doi:10.1111/cpr.70078)

Fig S1.

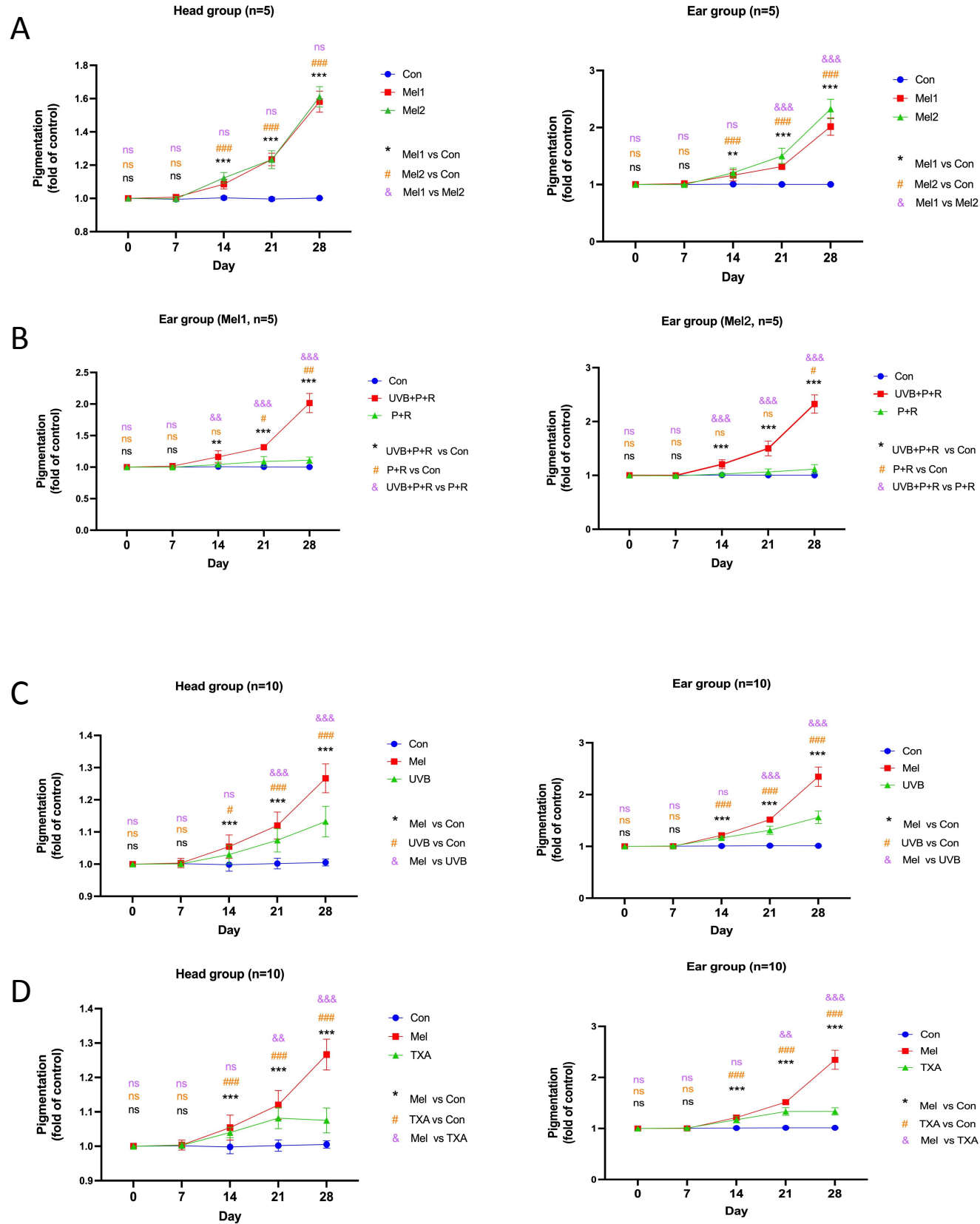

Fig S2.

A

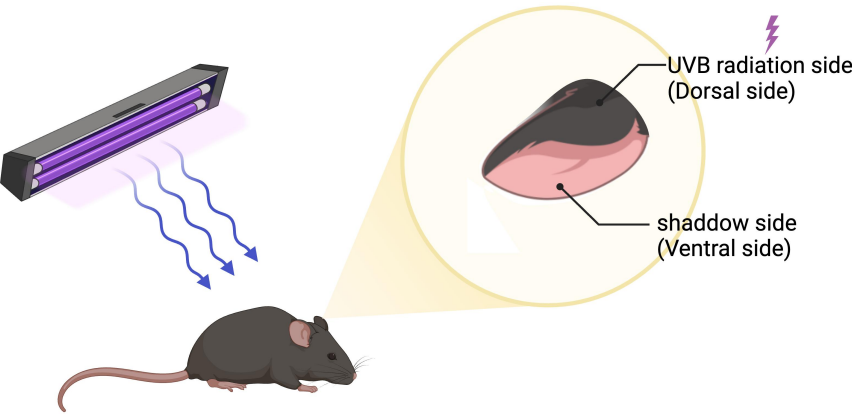

B

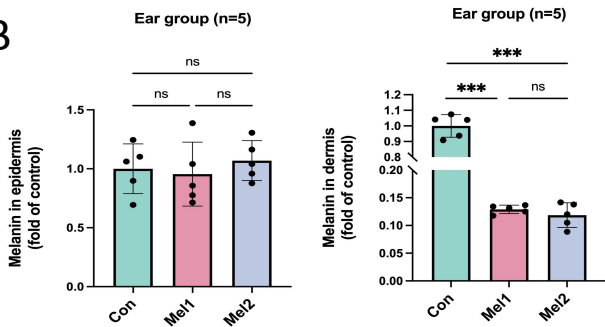

C

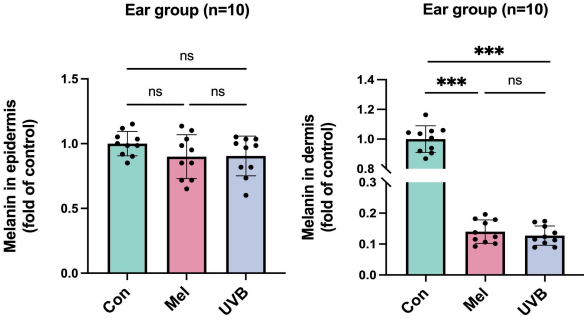

D

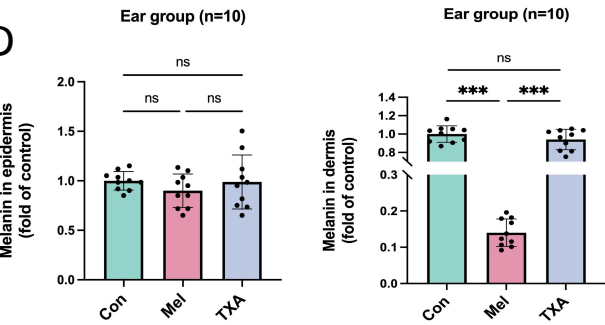

E

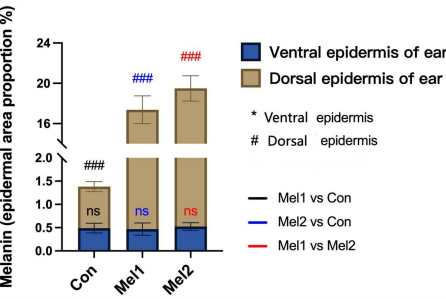

F

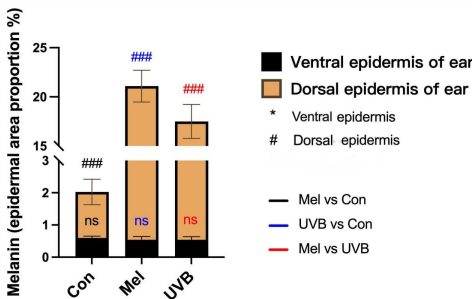

G

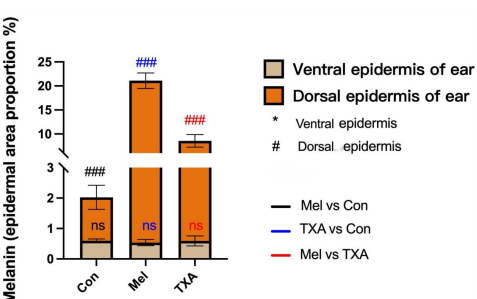

H

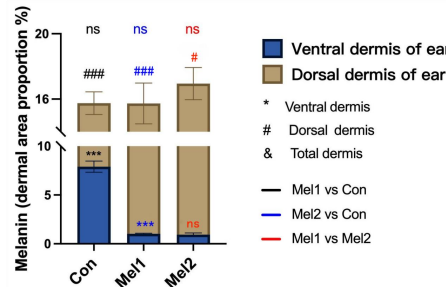

I

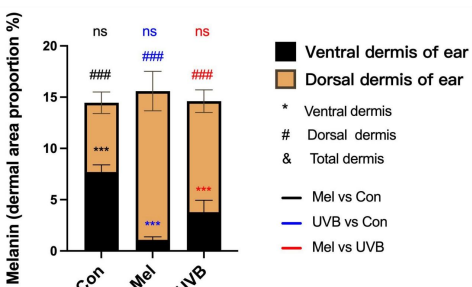

J

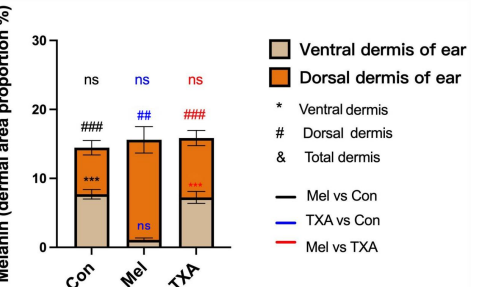

Supplement: Supplementary file 1 — Data S1. Supplementary Figure. [file CPR-59-e70078-s001.zip › cpr70078-sup-0001-Figures.pdf]
