# Supplementary material for: Establishment and Validation of a C57BL/6J Mouse Model for Melasma
Source: Cell Prolif. 2025 Jul 10;59(1):e70078. doi: 10.1111/cpr.70078 (PMC12774621; doi:10.1111/cpr.70078)
Supplement: Supplementary file 2 — Data S2. Supporting Information. [file CPR-59-e70078-s002.pdf]

实验动物伦理审查申请表

申请日期：　2023 年 10 月 13 日

受理编号：2023-DW-014

批准文号：2023 动物第014号.

项目名称	黄褐斑动物模型研究		
项目负责人	林彤	科室	激光科
邮箱	ddlin@hotmail.com	职称	主任医师
联系电话	13951902258		
动物实验负责人	王雯竹	职称	
联系电话	18292877056		

动物实验设施单位名称：中国医学科学院皮肤病医院（中国医学科学院皮肤病研究所）

许可证编号：SYXK（苏）2022-0017

参与动物实验操作人员姓名、相关培训证书及编号：王雯竹（编号：220223115）；孙小洁（编号：220221754）；杨荷丹（编号：）；张晓丽（编号：220221682）；李秀珍（编号：2022087）；金嘉轩（编号：220223661）。

拟实验时间：2023 年 10 月 28 日

动物替代、减少动物用量、降低动物痛苦伤害的主要措施

1. 研究拟在部分研究中利用离体培养的组织、细胞等代替整体动物实验进行研究分析，尽可能替代实验动物；

2. 降低动物痛苦伤害:在部分操作(涂抹药物、照射紫外线、图像采集等)前，采用 3%异氟烧吸入进行麻醉;小鼠的生物样本取材前，采用二氧化碳安乐死方式进行小动物人道处死；

3. 在小鼠背部皮肤紫外线照射实验中遮盖头部，避免伤伤害眼部。小鼠实验过程中，需要肌内注射药物时，注意轻柔操作，避免伤及周围组织；

4. 小鼠生物样本获取选择在专用的手术环境，远离饲养区域，避免和减轻对其他动物同伴的恐惧和伤害。

总之，在执行各项实验动物项目时，以人道的方式来管理及使用实验动物，并符合法规政策的要求，严格保护动物福利。

是否使用有毒（害）物质（感染、放射、化学毒、其他）

☑否

☐是，详细说明：

声明：

1、我将自觉遵守实验动物福利伦理相关法规和各项规定，同意接受伦理委员会的监督；

2、本人保证本申请表中所填内容真实、详尽和易懂。

项目负责人签字：[illegible]

日期：2023.10.17

动物实验负责人签字：[illegible]

日期：2023.10.13

伦理委员会审查意见：

☒同意

☐不同意

主任/副主任委员签字：[illegible]

日期：2023.10.27
